# Finite element analysis (FEA) of the stress behavior of some dental materials

**DOI:** 10.25122/jml-2025-0005

**Published:** 2025-01

**Authors:** Paolo Di Francesco, Anamaria Bechir, Anca Iuliana Popescu, Manuela Victoria Chivu, Anca Monica Dobrescu, Raluca Monica Comăneanu, Mihail Târcolea

**Affiliations:** 1 Doctoral School of Dental Medicine, Titu Maiorescu University, Bucharest, Romania; 2 Department of Specialized Dental Medicine Disciplines, Faculty of Dental Medicine, Titu Maiorescu University, Bucharest, Romania; 3 Doctoral School of Materials Science and Engineering, Politehnica University of Bucharest, Bucharest, Romania

**Keywords:** finite element analysis, zirconia, lithium disilicate, 3D-printed composites

## Abstract

This study compared the biomechanical behavior of three widely used dental materials—zirconia, lithium disilicate (IPS e.max CAD), and 3D-printed composite (VarseoSmile CrownPlus)— for maxillary anterior bridge restorations. Finite element analysis (FEA) was employed to evaluate the mechanical response of these materials under normal occlusal forces, replicating real clinical conditions. Key factors analyzed included stress distribution, deformation, and potential for failure under high loads. For each material, material constants such as Poisson’s ratio and Young’s modulus were used in the simulations, with the values chosen from validated literature sources. The findings demonstrated that zirconia exhibited superior mechanical strength and uniform stress distribution, making it an ideal material for posterior restorations subjected to high biomechanical stresses. Lithium disilicate showed balanced stress distribution and proved to be a versatile material suitable for both anterior and moderate-load restorations, with its superior aesthetic properties making it an attractive option for anterior areas. On the other hand, 3D-printed composite materials were found to have higher stress concentrations, particularly in occlusal regions, and exhibited lower elasticity compared to the other materials, limiting their use in permanent restorations but making them suitable for temporary restorations or areas with lower mechanical demands. This study provides valuable insights into the selection of dental materials for different clinical scenarios, emphasizing the importance of FEA in optimizing material choice and restoration design. The results suggest that while zirconia is ideal for long-term durability, lithium disilicate remains the preferred choice for aesthetic requirements, with 3D-printed composites serving as a promising alternative for long-term temporary applications.

## INTRODUCTION

Finite element analysis (FEA) is a highly advanced numerical technique widely employed across various engineering fields to analyze the mechanical behavior of complex structures. In dentistry, the application of FEA has become indispensable, significantly influencing the research and development of materials used in prosthetic restorations. Unlike traditional experimental methods, FEA is a non-invasive technique that enables the simulation of diverse clinical scenarios, providing reliable data for optimizing restoration design and material selection [1].

Recent advancements in CAD/CAM technology and 3D printing have revolutionized digital dental restorations, expanding treatment possibilities in modern dentistry. These technologies enable the production of customized restorations with high dimensional accuracy and enhanced mechanical properties [2]. FEA allows researchers to evaluate and compare the performance of restorative materials under controlled conditions, simulating the real mechanical stresses they undergo in the oral cavity [3].

FEA has confirmed that zirconia exhibits a uniform stress distribution even under significant occlusal loads [4]. This characteristic makes it an ideal material for use in the posterior regions of the dental arches, where mechanical stresses are intense and frequent. FEA-based studies have shown that IPS e.max CAD exhibits a balanced stress distribution, making it ideal for crowns and bridges subjected to moderate loads. The elastic properties of this material enable efficient stress absorption, reducing the risk of microfractures and ensuring optimal durability [5]. 3D-printed hybrid composite materials represent an innovative alternative for dental restorations due to their fast and efficient manufacturing process. 3D printing enables the production of customized restorations with minimal material consumption and precise adaptation to tooth morphology. However, FEA has identified certain limitations of these materials regarding mechanical strength [6]. Compared to zirconia and lithium disilicate-based ceramics, composite materials exhibit higher stress concentrations in occlusal contact areas and more pronounced deformations under high loads. This vulnerability is attributed to the lower modulus of elasticity of 3D-printed composite materials, which limits their use in permanent restorations exposed to intense biomechanical stresses. Nevertheless, these materials remain a viable solution for temporary restorations or applications where occlusal forces are minimal [7].

FEA provides an efficient approach to optimizing the design of complex restorations, such as anterior bridges. Parameters such as material thickness, connector geometry, and material type influence stress distribution and the overall durability of the structure. Studies have shown that adjusting these variables can reduce stress concentrations in critical areas, thereby extending the lifespan of the restoration [8].

The aim of this study was to perform a comparative analysis of the biomechanical behavior of three modern dental materials—zirconia, lithium disilicate-based ceramic (IPS e.max CAD), and 3D-printed composite (VarseoSmile Crown Plus)—for a maxillary anterior bridge using FEA.

## MATERIAL AND METHODS

This study was conducted at the National University of Science and Technology Politehnica Bucharest, Faculty of Materials Science and Engineering, within the BIOMAT Research Center. A three-dimensional model of a dental anterior bridge was processed using the Mimics Innovation Suite (Materialise N.V.), a specialized software that uses CBCT data to generate accurate anatomical reconstructions. The model was discretized into tetrahedral elements to ensure an accurate representation of its geometry and mechanical behavior. This process involved the application of multiple segmentation masks to enhance anatomical detail, followed by the generation of the surface mesh and the final volumetric structure.

**Figure 1:**
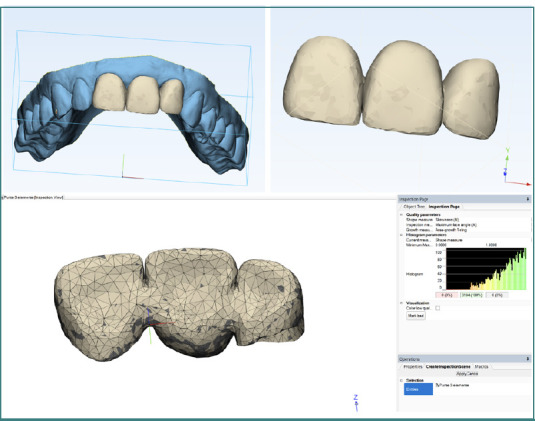
Three-dimensional model of the dental bridge discretized using 3-matic^©^ Materialise N.V. software

The maxillary anterior bridge represents a complex restoration with high biomechanical and aesthetic requirements. The three-dimensional STL model of the bridge was discretized using the same advanced software, Mimics, and 3-matic Materialise N.V., to ensure an accurate and detailed representation of the structure. The discretization of the three-dimensional model (Figure 1) highlighted the importance of using a high-quality finite mesh in FEA analyses.

Element size selection and geometry-based optimization ensured precise, clinically relevant results. This process emphasized the importance of meticulous attention during the preprocessing stage to ensure the validity of the results and their applicability in modern prosthetic practice.

**Figure 2:**
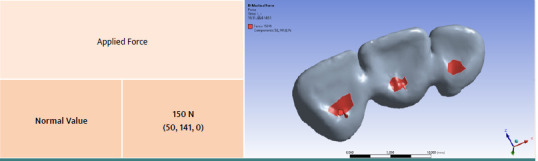
Applied force on contact surfaces and fixed support on the two pillars of the maxillary bridge

For a realistic and accurate simulation, contact points must be placed in accordance with occlusal principles and a normal occlusal scheme. These principles ensure the proper distribution of occlusal forces. The placement of the contact points was performed following the fundamental rules of functional occlusion. The contact points were positioned on the lingual surface of the upper central incisors and lateral incisors near the cingulum, simulating maximum intercuspation in centric relation. A standard occlusal force of 150 N was applied in the simulation (Figure 2).

In this study, the stresses and deformations within the bridge were analyzed for three materials commonly used in prosthetic restorations: zirconium oxide (Zirkon BioStar Ultra), lithium disilicate (IPS e.max CAD), and 3D-printed composite (VarseoSmile Crownplus). The simulations required the input of material constants, including Poisson’s ratio and Young’s modulus, which were selected based on validated values from the scientific literature [9-15] (Table 1).

**Table 1:** Properties of materials used in the fabrication of the restoration

	Young's Modulus (MPa)	Poisson's Ratio (-)
Zirkon BioStar Ultra	2,0 x 10^5^	0,31 - 0,33
IPS e.max CAD	8,35 x 10^4^	0,21 - 0,25
VarseoSmile Crownplus	4,03 x 10^3^	0,25 - 0,35

The prosthetic component was analyzed under point loads applied to the previously defined contact areas. The three-dimensional STL model, discretized using 3-matic Materialise N.V. software, was imported into the ANSYS Workbench environment.

## RESULTS

When applying the maximum normal force of 150 N to the analyzed dental bridge, the force was decomposed into three directions as follows: 50 N along the OX axis, 141 N along the OY axis, and 0 N along the OZ axis. For each material, the following parameters were calculated: total deformation, deformation along the OX, OY, and OZ axes, equivalent elastic stress, maximum, mean, and minimum principal elastic stress, maximum elastic shear stress, equivalent stress, maximum, mean, and minimum principal stress, maximum shear stress, normal stress along the OX, OY, and OZ axes, and shear stress along the XY, YZ, and XZ planes.

The maximum, minimum, and mean stress and deformation values are summarized in the tables below for each material used in the dental bridge (Tables 2a, 2b, 3a, 3b, 4a, 4b). Additionally, the tables specify the areas on the crowns where extreme stresses and deformations developed, highlighting potentially vulnerable zones during masticatory cycles or parafunctional activities.

**Table 2a:** Force of 150 N – Zircon Biostar Ultra bridge

	Minimum	Maximum	Average	Minimum Occurs	OnMaximum Occurs On
Results					
Total Deformation (mm)	0	2.2e-004	3.01e-005	2.2 in the distal marginal ridge’s lower area	Cingulum of 2.1
Directional Deformation X (mm)	-2.13e-005	5.76e-005	4.17e-006	Distal to the cingulum of 2.1	Mesial to the cingulum of 2.1
Directional Deformation Y (mm)	-2.51e-005	2.12e-004	1.55e-005	Disto-incisal angle of 2.1	Cingulum of 2.1
Directional Deformation Z (mm)	-9.04e-005	1.7e-004	7.31e-006	Central area of the incisal edge of 2.1	Above the cingulum of 2.1
Equivalent Elastic Strain (mm/mm)	1.44e-009	7.84e-005	6.48e-006	Below the distal contact point of 1.1	Cingulum of 1.1
Maximum Principal Elastic Strain (mm/mm)	5.97e-010	4.7e-005	3.99e-006	Below the distal contact point of 1.1	Cingulum of 1.1
Middle Principal Elastic Strain (mm/mm)	-1.61e-005	1.4e-005	-2.41e-008	Cingulum of 2.1	Above the cingulum of 2.1
Minimum Principal Elastic Strain (mm/mm)	-7.77e-005	-4.54e-010	-4.53e-006	Cingulum of 1.1	Below the distal contact point of 1.1
Maximum Shear Elastic Strain (mm/mm)	1.38e-009	1.17e-004	8.52e-006	Below the distal contact point of 1.1	Cingulum of 1.1
Equivalent Stress (MPa)	1.96e-004	16.3	1.21	Under the distal contact point of 1.1	Cingulum of 1.1

**Table 2b:** Force of 150 N – Zircon Biostar Ultra bridge

	Minimum	Maximum	Average	Minimum Occurs	OnMaximum Occurs On
Results					
Maximum Principal Stress (MPa)	-5.26	9.58	0.542	Cingulum of 2.2	Below the contact point between 1.1 and 2.1
Middle Principal Stress (MPa)	-8.36	1.62	-9.14e-002	Cingulum of 2.1	Below the contact point between 1.1 and 2.1
Minimum Principal Stress (MPa)	-18.4	0.726	-0.803	Cingulum of 2.1	Cingulum of 1.1
Maximum Shear Stress (MPa)	1.09e-004	9.22	0.673	Below the distal contact point of 1.1	Cingulum of 1.1
Normal Stress X (MPa)	-10.4	5.13	-2.77e-002	Cingulum of 2.1	Distal to the cingulum of 1.1
Normal Stress Y (MPa)	-17	5.91	-0.314	Cingulum of 1.1	Below the contact point between 2.1 and 2.2
Normal Stress Z (MPa)	-6.33	5.07	-1.11e-002	Cingulum of 2.1	Cingulum of 1.1
Shear Stress XY (MPa)	-5.02	3.99	-5.55e-002	Cingulum of 2.1	Below the contact point between 1.1 and 2.1
Shear Stress YZ (MPa)	-3.8	4.66	2.38e-002	Cingulum of 2.1	Above the cingulum of 2.1
Shear Stress XZ (MPa)	-4.22	2.67	-7.e-002	Cingulum of 1.1	Below the contact point between 1.1 and 2.1

**Table 3a:** Force of 150 N – IPS e.max CAD bridge

	Minimum	Maximum	Average	Minimum Occurs	OnMaximum Occurs On
Results					
Total Deformation (mm)	0	5.46e-004 mm	7.24e-005	2.2 in the lower area of the distal marginal ridge	Cingulum of 2.1
Directional Deformation X (mm)	-5.39e-005	1.45e-004	1.05e-005	Distal to the cingulum of 2.1	Mezial to the cingulum of 2.1
Directional Deformation Y (mm)	-5.86e-005	5.26e-004	3.77e-005	Disto-incisal angle of 2.1	Cingulum of 2.1
Directional Deformation Z (mm)	-2.14e-004	4.08e-004	1.79e-005	Central area of the incisal edge of 2.1	Above the cingulum of 2.1
Equivalent Elastic Strain (mm/mm)	2.27e-009	2.04e-004	1.63e-005	Below the distal contact point of 1.1	Cingulum of 1.1
Maximum Principal Elastic Strain (mm/mm)	1.e-009	1.17e-004	9.23e-006	Below the distal contact point of 1.1	Cingulum of 1.1
Middle Principal Elastic Strain (mm/mm)	-5.66e-005	2.68e-005	-1.82e-007	Cingulum of 2.1	Above the cingulum of 2.1
Minimum Principal Elastic Strain (mm/mm)	-2.e-004	-8.28e-010	-1.09e-005	Cingulum of 1.1	Below the distal contact point of 1.1
Maximum Shear Elastic Strain (mm/mm)	2.53e-009	2.85e-004	2.01e-005	Below the distal contact point of 1.1	Cingulum of 1.1
Equivalent Stress (MPa)	1.49e-004	16.9	1.21	Below the distal contact point of 1.1	Cingulum of 1.1

**Table 3b:** Force of 150 N – IPS e.max CAD bridge

	Minimum	Maximum	Average	Minimum Occurs	OnMaximum Occurs On
Results					
Maximum Principal Stress (MPa)	-4.34	9.2	0.555	Cingulum of 2.2	Below the contact point between 1.1 and 2.1
Middle Principal Stress (MPa)	-8.47	1.36	-7.36e-002	Cingulum of 2.1	Mezial to the cingulum of 2.2
Minimum Principal Stress (MPa)	-17.7	0.483	-0.788	Cingulum of 1.1	Cingulum of 1.1
Maximum Shear Stress (MPa)	8.45e-005	9.5	0.672	Below the distal contact point of 1.1	Cingulum of 1.1
Normal Stress X (MPa)	-9.24	5.43	-7.34e-003	Cingulum of 2.1	Distal to the cingulum of 1.1
Normal Stress Y (MPa)	-16.4	5.66	-0.305	Cingulum of 1.1	Below the contact point between 2.1 and 2.2
Normal Stress Z (MPa)	-6.08	5.17	5.74e-003	Cingulum of 2.1	Cingulum of 1.1
Shear Stress XY (MPa)	-5.01	3.98	-5.65e-002	Cingulum of 2.1	Below the contact point between 1.1 and 2.1
Shear Stress YZ (MPa)	-4.1	4.68	1.79e-002	Cingulum of 2.1	Above the cingulum of 2.1
Shear Stress XZ (MPa)	-4.48	2.71	-6.89e-002	Cingulum of 1.1	Below the contact point between 1.1 and 2.1

**Table 4a:** Force of 150 N – VarseoSmile CAD bridge

	Minimum	Maximum	Average	Minimum Occurs	OnMaximum Occurs On
Results					
Total Deformation (mm)	0	1.15e-002	1.59e-003	2.2 in the lower area of the distal marginal ridge	Cingulum of 2.1
Directional Deformation X (mm)	-1.11e-003	3.e-003	2.17e-004	Distal to the cingulum of 2.1	Mezial to the cingulum of 2.1
Directional Deformation Y (mm)	-1.33e-003	1.11e-002	8.13e-004	Disto-incisal angle of 2.1	Cingulum of 2.1
Directional Deformation Z (mm)	-4.78e-003	8.97e-003	3.83e-004	Central area of the incisal edge of 2.1	Above the cingulum of 2.1
Equivalent Elastic Strain (mm/mm)	8.43e-008	4.05e-003	3.37e-004	Below the distal contact point of 1.1	Cingulum of 1.1
Maximum Principal Elastic Strain (mm/mm)	3.62e-008	2.46e-003	2.12e-004	Below the distal contact point of 1.1	Cingulum of 1.1
Middle Principal Elastic Strain (mm/mm)	-8.05e-004	7.95e-004	-5.71e-007	Cingulum of 2.1	Above the cingulum of 2.1
Minimum Principal Elastic Strain (mm/mm)	-4.01e-003	-2.31e-008	-2.39e-004	Cingulum of 1.1	Below the distal contact point of 1.1
Maximum Shear Elastic Strain (mm/mm)	6.75e-008	6.13e-003	4.51e-004	Below the distal contact point of 1.1	Cingulum of 1.1
Equivalent Stress (MPa)	1.81e-004	16.2	1.21	Below the distal contact point of 1.1	Cingulum of 1.1

**Table 4b:** Force of 150 N – VarseoSmile CAD bridge

	Minimum	Maximum	Average	Minimum Occurs	OnMaximum Occurs On
Results					
Maximum Principal Stress (MPa)	-5.5	9.69	0.539	Cingulum of 2.2	Below the contact point between 1.1 and 2.1
Middle Principal Stress (MPa)	-8.29	1.74	-9,66e-002	Cingulum of 2.1	Below the contact point between 1.1 and 2.1
Minimum Principal Stress (MPa)	-18.7	0.764	-0.808	Cingulum of 2.1	Cingulum of 1.1
Maximum Shear Stress (MPa)	1,01e-004	9.15	0.673	Below the distal contact point of 1.1	Cingulum of 1.1
Normal Stress X (MPa)	-10.8	5.04	-3,35e-002	Cingulum of 2.1	Distal to the cingulum of 1.1
Normal Stress Y (MPa)	-17.2	5.99	-0.317	Cingulum of 1.1	Below the contact point between 2.1 and 2.2
Normal Stress Z (MPa)	-6.39	5.04	-1,57e-002	Cingulum of 2.1	In the cingulum of 1.1
Shear Stress XY (MPa)	-5.02	4	-5,52e-002	Cingulum of 2.1	Below the contact point between 1.1 and 2.1
Shear Stress YZ (MPa)	-3.72	4.65	2,55e-002	Cingulum of 2.1	Above the cingulum of 2.1
Shear Stress XZ (MPa)	-4.15	2.68	-7,03e-002	Cingulum of 1.1	Below the contact point between 1.1 and 2.1

## DISCUSSION

The use of FEA was essential for understanding how each material responds to mechanical loads. Zirconia exhibited superior biomechanical performance, with uniform stress distribution and high fracture resistance under mechanical loads. The FEA results showed that zirconium oxide exhibits favorable stress distribution in critical areas, preventing deformations and localized stress accumulations, which contributes to the long-term stability of restorations [16,17]. In contrast, lithium disilicate combines superior aesthetics due to its translucency, which mimics natural enamel, and its adequate mechanical strength, making it a common choice for restorations in both anterior and posterior regions, depending on clinical requirements. Studies indicate that this material is ideal for restorations requiring both mechanical strength and high aesthetics [18-20]. Although 3D-printed composites offer advantages in manufacturing flexibility and speed, they have limitations when exposed to high biomechanical stresses. These materials are more suitable for temporary restorations or those exposed to moderate mechanical loads [19-22].

By applying FEA, this study successfully identified the critical points in the behavior of each material, highlighting their respective contributions to optimizing restoration design. This method enabled the simulation of real clinical scenarios and the assessment of each material’s suitability for specific applications, thereby providing a valuable contribution to future approaches in restorative dentistry.

Park *et al*. [23] highlighted in a detailed study that 3D-printed composite materials are primarily suitable for temporary restorations. Their lower modulus of elasticity contributes to a limited capacity to withstand intense mechanical forces, making them more prone to deformations and fractures. In comparison, materials manufactured through subtractive technologies, such as zirconia and lithium disilicate, demonstrated superior performance under similar loading conditions.

The results obtained in this study align with the conclusions of existing literature. Our finite element analysis revealed that the 3D-printed composite, VarseoSmile CrownPlus, exhibited significant limitations under biomechanical loading conditions. Specifically, the material showed a tendency to concentrate stresses in critical areas, which could compromise the structural integrity of the restoration over time. Nevertheless, its performance is adequate for temporary restorations or regions with moderate biomechanical demands, providing a viable alternative in certain clinical contexts.

Although 3D-printed composite materials exhibit limitations in mechanical strength, recent studies, such as that by Tribst *et al*. [24], have demonstrated that innovative techniques, such as preheating the restorative material and the cementing agent, can enhance mechanical properties and interfacial adhesion. These methods could represent a viable solution for improving the biomechanical performance of 3D-printed restorations, especially under conditions of intense occlusal loading.

The results obtained in this study align with the in vitro validations conducted by Waldecker *et al*. [16], which confirmed the durability of zirconia in complex clinical applications. Their study demonstrated that zirconia remains stable and functional even under intense mechanical stresses, making it an excellent choice for long-term restorations.

Lithium disilicate (IPS e.max CAD) is particularly preferred for restorations in anterior regions, where esthetic demands are high. The mechanical properties of this material ensure efficient stress absorption, thereby reducing the risk of microfractures. Hofsteenge *et al*. [19] emphasized these aspects, demonstrating that lithium disilicate provides balanced biomechanical performance, making it ideal for esthetic and functional requirements.

Another essential aspect of the performance of lithium disilicate is its specific layering, which enhances its biomechanical behavior. Rodrigues *et al*. [25] demonstrated that the use of this material in bilayer restorations reduces stress concentrations, uniformly distributing occlusal loads and preventing premature damage to the restoration.

The results obtained in this study are also consistent with those reported by El-Farag *et al*. [26], who confirmed that lithium disilicate offers superior mechanical strength, making it suitable for restorations exposed to moderate loads.

Atria *et al*. [21] highlighted that 3D-printed materials, although effective for temporary restorations, exhibit lower mechanical strength compared to materials manufactured using subtractive techniques. This finding is further supported by Park *et al*. [23], who demonstrated that the reduced elastic modulus limits the use of these materials in permanent restorations. Our study corroborates these observations, showing higher stress concentrations in the occlusal regions of 3D-printed composites, consistent with the findings of Alshamrani *et al*. [27] regarding the behavior of 3D-LCM materials under high-load conditions.

The findings of our study regarding the limitations of 3D-printed composite materials are supported by Baciu *et al*. [28], who demonstrated the inferiority of these materials compared to those obtained through subtractive processing, both in terms of mechanical strength and stress behavior. The FEA analysis conducted in their study corroborates our conclusions about stress distribution and the role of optimized design in reducing critical stress concentrations in prosthetic restorations.

Finite element analysis continues to play a crucial role in evaluating biomechanical behavior across different dental applications, from prosthetic stability to orthodontic force distribution. Reddy *et al*. demonstrated that subcrestal placement and platform switching in dental implants contribute to stress reduction in D3 bone, with implant diameter playing a more critical role than implant length in mitigating stress concentrations [29]. This finding underscores the importance of implant design optimization, aligning with our study’s focus on material selection and its role in stress distribution in maxillary anterior restorations.

Similarly, Mańkowski *et al*. developed a high-fidelity FEM model of the mandible with the temporomandibular joint to evaluate the strength and fatigue properties of bonding elements used in fracture and defect surgeries. Their research highlights the necessity of accurate anatomical modeling, boundary conditions, and meshing strategies to ensure biomechanical validity in FEM simulations [30]. This aligns with our approach to evaluating material stress distribution, further reinforcing the role of computational models in predicting long-term stability and optimizing prosthetic designs under dynamic loading conditions.

Kendre *et al*. investigated stress changes in the maxilla due to fixed functional appliances, revealing a significant increase in von Mises and principal stresses, particularly in the posterior maxilla [31]. Their findings demonstrated how applied forces can lead to stress accumulation, similar to our observations regarding stress variations in different restorative materials. This highlights the importance of selecting materials capable of withstanding complex loading conditions to ensure long-term biomechanical stability.

Further supporting the role of FEA in prosthetic optimization, Cervino *et al*. evaluated the biomechanical performance of the OT Bridge system and OT Equator retention elements in full-arch fixed prostheses, confirming that Seeger retention significantly enhances prosthetic stability [32].

Nagib *et al*. explored the mechanical behavior of individualized 3D-printed polymeric surgical guides for orthodontic mini-implant insertion, demonstrating their controlled displacement and safe stress levels [33]. Their results align with our findings on material-dependent stress distribution, emphasizing the benefits of CAD-based design in optimizing dental treatment precision. Likewise, Jindal *et al*. compared the mechanical behavior of 3D-printed and thermoformed aligners under non-linear compressive loading, confirming that 3D-printed aligners exhibit superior dimensional accuracy while maintaining mechanical strength [34].

These studies validate the role of FEM in optimizing dental material selection, force application, and long-term prosthetic performance. The continued advancements in computational modeling provide valuable insights into how different materials behave under varying mechanical stresses, reinforcing the need for precise material selection, design optimization in clinical practice, and the growing role of digital workflows in enhancing material selection and biomechanical performance in prosthetic, surgical and orthodontic applications.

The findings of this study provide essential clinical insights into long-term prosthetic performance under varying loading conditions. Material selection plays a crucial role in ensuring restoration longevity, particularly in high-stress areas. Zirconia remains the preferred material for restorations subjected to intense occlusal forces due to its high fracture resistance and stress distribution properties. Lithium disilicate provides a balance between mechanical strength and esthetics, making it suitable for anterior restorations with moderate load-bearing requirements. Meanwhile, 3D-printed composites, while promising for temporary restorations, require further mechanical reinforcement to enhance their performance in permanent applications. Clinicians must carefully consider occlusal loading conditions, patient-specific factors, and prosthetic design to optimize long-term outcomes and minimize failure risks.

## CONCLUSION

This study provided a detailed analysis of the biomechanical behavior of three materials—zirconia, lithium disilicate (IPS e.max CAD), and 3D-printed composite (VarseoSmile CrownPlus)—using FEA. The results, compared with existing literature, led to the following conclusions:

- Zirconia demonstrated superior mechanical strength and uniform stress distribution.
- Lithium disilicate (IPS e.max CAD) was a versatile material with remarkable biomechanical adaptability. FEA analyses revealed balanced stress distribution, making it suitable for anterior restorations and those subjected to moderate loads.
- 3D-printed composite materials showed high-stress concentrations in occlusal areas and reduced elasticity compared to zirconia and lithium disilicate, limiting their use in permanent restorations exposed to high biomechanical loads. Despite their mechanical limitations, the efficient manufacturing process and design flexibility make 3D-printed composites a viable option for temporary restorations.

The use of FEA allowed for a detailed examination of stress distribution and material behavior under clinically relevant conditions. This study enhances the understanding of modern dental material performance and offers valuable insights for practitioners in selecting materials based on clinical requirements.
